# The transcription factor WRKY75 positively regulates jasmonate-mediated plant defense to necrotrophic fungal pathogens

**DOI:** 10.1093/jxb/eraa529

**Published:** 2020-11-09

**Authors:** Ligang Chen, Liping Zhang, Shengyuan Xiang, Yanli Chen, Haiyan Zhang, Diqiu Yu

**Affiliations:** 1 CAS Key Laboratory of Tropical Plant Resources and Sustainable Use, Xishuangbanna Tropical Botanical Garden, Chinese Academy of Sciences, Mengla, Yunnan, China; 2 Center of Economic Botany, Core Botanical Gardens, Chinese Academy of Sciences, Menglun, Mengla, Yunnan, China; 3 CAS Key Laboratory for Plant Diversity and Biogeography of East Asia, Kunming Institute of Botany, Chinese Academy of Sciences, Kunming, Yunnan, China; 4 University of Chinese Academy of Sciences, Beijing, China; 5 Bielefeld University, Germany

**Keywords:** *Botrytis cinerea*, JA-associated genes, jasmonate, JAZ, necrotrophic pathogen, WRKY75

## Abstract

Necrotrophic fungi cause devastating diseases in both horticultural and agronomic crops, but our understanding of plant defense responses against these pathogens is still limited. In this study, we demonstrated that WRKY75 positively regulates jasmonate (JA)-mediated plant defense against necrotrophic fungal pathogens *Botrytis cinerea* and *Alternaria brassicicola,* and also affects the sensitivity of plants to JA-inhibited seed germination and root growth. Quantitative analysis indicated that several JA-associated genes, such as OCTADECANOID-RESPONSIVE ARABIDOPSIS (*ORA59*) and PLANT DEFENSIN 1.2A (*PDF1.2*), were significantly reduced in expression in *wrky75* mutants, and enhanced in *WRKY75* overexpressing transgenic plants. Immunoprecipitation assays revealed that WRKY75 directly binds to the promoter of *ORA59* and represses itstranscription. *In vivo* and *in vitro* experiments suggested that WRKY75 interacts with several JASMONATE ZIM-domain proteins, repressors of the JA signaling pathway. We determined that JASMONATE-ZIM-DOMAIN PROTEIN 8 (JAZ8) represses the transcriptional function of WRKY75, thereby attenuating the expression of its regulation. Overexpression of *JAZ8* repressed plant defense responses to *B. cinerea*. Our study provides evidence that WRKY75 functions as a critical component of the JA-mediated signaling pathway to positively regulate Arabidopsis defense responses to necrotrophic pathogens.

## Teaser

The transcription factor WRKY75 functions in the jasmonate signaling pathway to modulate plant defense responses against necrotrophic fungal pathogens.

## Introduction

As sessile organisms, plants constantly encounter various fluctuating environmental stresses, including attacks from microbial pathogens and herbivores. As a result of these long-term, constant biotic interactions, resistant plants have successfully evolved sophisticated defense mechanisms for protection. In addition to constitutive physical and chemical strategies, plants typically use a powerful inducible defense system to fend off various attackers (Jones and Dangl, 2006; Howe and Jander, 2008; Ramirez-Prado *et al.*, 2018). To enhance disease resistance, the plant defense system largely depends on the inducible expression of numerous host defense-related genes. Constitutive and inducible defense systems together constitute a multi-layered network that can be initiated sequentially in response to pathogen or herbivore attack (Nishimura and Dangl, 2010; van der Burgh and Joosten, 2019).

Plant defense responses upon pathogen infection are the consequence of highly coordinated, sequential changes at the cellular level in which plant hormones play important roles. Numerous studies have demonstrated that salicylic acid (SA), jasmonate (JA), and ethylene (ET) are the primary defense hormones. The importance of these hormones in plant innate immunity is well documented, particularly in the model plant *Arabidopsis thaliana* (Grant and Jones, 2009; Robert-Seilaniantz *et al.*, 2011). The SA signaling pathway is mainly linked to resistance against biotrophic pathogens, which feed on living host tissues, and are often associated with hypersensitive response (Durrant and Dong, 2004; Vlot *et al.*, 2009; Ding and Ding, 2020); while the JA and ET signaling pathways are predominantly associated with resistance to necrotrophic pathogens that promote host cell death at early stages of infection (Glazebrook, 2005; Song *et al.*, 2014). Genetic and molecular analyses have demonstrated that there is extensive cross-talk between SA- and JA/ET-mediated defense signaling pathways in a synergistic or antagonistic manner (Thaler *et al.*, 2012).

Necrotrophic pathogens, such as *Botrytis cinerea*, *Alternaria brassicicola*, *Fusarium oxysporum*, and *Sclerotinia sclerotiorum*, cause serious devastating diseases in both horticultural and agronomic crops. Nevertheless, little is known about plant defense responses against these fungi. Compared with the well-known gene-for-gene resistance to numerous biotrophic pathogens, specific recognition of necrotrophic pathogens by host resistance proteins is uncommon. Indeed, the defense response of Arabidopsis to the necrotrophic pathogen *B. cinerea* seems to be under complex genetic control (Veloso and van Kan, 2018). Molecular and genetic studies have identified several genes and products that are involved in plant resistance to necrotrophic pathogens, such as RESISTANCE TO LEPTOSPHAERIA MACULANS 3 (RLM3) (Staal *et al.*, 2008), BOTRYTIS-INDUCED PROTEIN KINASE 1 (BIK1; Veronese *et al.*, 2006), MITOGEN-ACTIVATED PROTEIN KINASE3 (MAPK3; Ren *et al.*, 2008), several autophagy genes (Lai *et al.*, 2011b), enzymes of cutin biosynthesis and secondary cell wall formation (Hernández-Blanco *et al.*, 2007; Serrano *et al.*, 2014), and some secondary metabolites, including glucosinolates, camalexin, and phenolic compounds (Kliebenstein *et al.*, 2005; Ahuja *et al.*, 2012; Burow and Halkier, 2017). In addition, global transcriptional profiling studies have demonstrated that infection by necrotrophic pathogens results in massive transcriptional reprogramming in the host, thereby indicating the involvement of certain transcription factors in this process (Abu-Qamar *et al.*, 2006; Birkenbihl *et al.*, 2017). As expected, several transcription factors, such as MYC2, ETHYLENE INSENSITIVE 3 (EIN3), MYB transcription factors (MYB46 and MYB108), ORA59, and ETHYLENE RESPONSE FACTOR 1 (ERF1), have been identified to be involved in plant defense against necrotrophic pathogens (Berrocal-Lobo *et al.*, 2002; Mengiste *et al.*, 2003; Lorenzo *et al.*, 2004; Pré*et al*., 2008; Ramírez *et al.*, 2011). In spite of these studies, our understanding of plant defense against necrotrophic pathogens is still limited.

The phytohormone JA acts as an important regulatory signal to control multiple plant processes, such as root growth, plant fertility, tuberization, anthocyanin accumulation, senescence, fruit ripening, and defense responses (Pauwels and Goossens, 2011). Studies have demonstrated that JA is perceived by the F-box protein CORONATINE INSENSITIVE1 (COI1), which subsequently facilitates the ubiquitination and degradation of JASMONATE-ZIM-DOMAIN (JAZ ) proteins via the CORONATINE INSENSITIVE1 (COI1)–based Skp1/Cullin/F-box complex (SCF^COI1^)-26S proteasome pathway (Chini *et al.*, 2007; Thines *et al.*, 2007; Yan *et al.*, 2007; Sheard *et al.*, 2010). JAZ family of proteins function as repressors of the JA signaling pathway via their physical interactions with a wide array of transcription factors. Degradation of JAZ proteins lead to the release and activation of various transcription factors which subsequently regulate downstream signaling cascades and modulate their respective JA responses (Pauwels and Goossens, 2011). Several key transcription factors have recently been identified as direct targets of JAZ proteins. For example, basic helix-loop-helix (bHLH) subgroup IIId transcription factors (bHLH3, bHLH13, bHLH14, and bHLH17) and bHLH subgroup IIIe transcription factors (MYC2, MYC3, and MYC4)function as direct targets of JAZ proteins to regulate JA-mediated plant defense and development (Niu *et al.*, 2011; Cheng *et al.*, 2011; Fernández-Calvo *et al.*, 2011; Song *et al*, 2013). R2R3-MYB transcription factors (MYB21, MYB24 and MYB57), essential components of WD-repeat/bHLH/MYB transcriptional complexes (TRANSPARENT TESTA 8 [TT8], GLABRA 3 [GL3], ENHANCER OF GLABRA 3 [EGL3], R2R3 MYB transcription factors [MYB75 and Glabra1]), bHLH transcription factors (INDUCER OF CBF EXPRESSION 1[ICE1], ICE2, and ROOT HAIR DEFECTIVE 6 [RHD6]), and APETALA2 transcription factors (TARGET OF EAT1 [TOE1] and TOE2) also interact with JAZ proteins to regulate JA-mediated male fertility, anthocyanin accumulation, trichome initiation, freezing tolerance, root hair growth, and flowering (Cheng *et al.*, 2009; Song *et al.*, 2011; Qi *et al.*, 2011; Hu *et al.*, 2013; Zhai *et al.*, 2015; Han *et al.*, 2020).

Although the WRKY family of transcription factors was shown to widely modulate host defenses toward various phytopathogens (Rushton *et al.*, 2010), the molecular mechanisms underlying their roles in plant defense responses remain to be further elucidated. WRKY75 was previously reported to participate in diverse biological processes, especially stress responses such as phosphate deficiency, root hair development, oxalic acid stress resistance, defense responses, and the unfolded protein response (Devaiah *et al.*, 2007; Li *et al.*, 2012; Chen *et al.*, 2013b; Rishmawi *et al.*, 2014; Schmiesing *et al.*, 2016). Recent studies further demonstrated that WRKY75 functions as a positive regulator during leaf senescence thorough promotion of SA biosynthesis and suppression of H_2_O_2_ scavenging, and also positively regulates flowering through a gibberellin (GA)-mediated signaling pathway (Guo *et al*, 2017; Zhang *et al.*, 2018).

Here, we used a molecular and genetic approach to demonstrate that altered expression of the *WRKY75* gene affects JA-regulated plant defense against necrotrophic pathogens. We showed that WRKY75 acts as a transcriptional activator to transmit JA-mediated plant defense signaling by directly binding to downstream target sequences such as *ORA59*. Moreover, we found that several JAZ proteins physically interact with WRKY75 and repress its transcriptional function, and that overexpression of *JAZ8* represses plant defense response to *B.cinerea*. Our results thus provide compelling evidence that WRKY75 functions as a positive regulator in JA-mediated defense response in Arabidopsis.

## Materials and methods

### Plant material and growth conditions

*Arabidopsis thaliana* plants were grown in an artificial growth chamber at 22°C with a 10h light/14h dark photoperiod. Columbia-0 (Col) was used as the wild type. We obtained *jaz8* mutants from the Arabidopsis Biological Resource Center (ABRC). The following Arabidopsis lines were used in this study: *WRKY75 RNAi* (Devaiah *et al.*, 2007), *wrky75-1*(SALK_101367; Lei *et al.*, 2014), *wrky75-25* (Encinas-Villarejo *et al.*, 2009; Rishmawi *et al.*, 2014), and*jaz8* (Jiang *et al.*, 2014). Plants harboring the *WRKY75:GUS:3’-WRKY75* were used in GUS staining experiments and plants harboring *WRKY75: YFP-WRKY75:3’-WRKY75* in *wrky75-25* background were used to observe YFP fluorescence (Rishmawi *et al.*, 2014).The plant materials *ORA59:GUS*, *35S:ORA59*and *35S:JAZ8-L8* were used as female parents in genetic analysis, while *WRKY75RNAi*, *wrky75-1* and *35S:WRKY75-L5* were used as male parents.

### Induction treatments

SA was dissolved in water as a 100 mM-stock solution and adjusted to pH 6.5 with KOH. Plants were sprayed with a 2 mM SA solution diluted from the stock. Methyl jasmonate (MeJa) was dissolved in 50% ethanol as a 10 mM stock solution. The MeJA stock solution was diluted to 100 μM with water and sprayed onto plants. Aminocyclopropane-1-carboxylic acid (ACC) was dissolved in water, and a 2 mM solution was sprayed onto plants.In all cases, water was sprayed onto plants as controls and the aerial parts of four week-old plants grown in soil were used.

### Expression analysis

For qRT–PCR analysis, total RNA was extracted using TRIzol reagent (Invitrogen, USA) and was treated with RNase-free DNase, according to the manufacturer’s instructions. Total RNA (1 μg) was reversetranscribed in a 20 μl reaction mixture using Superscript II (Invitrogen, USA). After the reaction, 1 μl aliquots were used as templates for qRT–PCR. Half reactions (10 μl each) were performed with the Light cycler Fast Start DNA Master SYBR Green I Kit (Roche, Mannheim, Germany) on a Roche light cycler 480 real-time PCR machine, according to the manufacturer’s instructions. *ACT2* (AT3G18780) and *UBQ5* (AT3G62250) were used as controls in quantitative RT–PCR. Analysis was conducted following the minimum information for publication of quantitative Real-Time PCR experiments guidelines (Bustin *et al.*, 2009; Supplementary Table S1). The gene-specific primers are listed in Supplementary Table S2.

For northern blot analyses, total RNA was extracted using TRIzol reagent. Approximately 20 μg RNA was separated on an agarose-formaldehyde gel and then blotted onto nylon membranes following standard procedures. The membranes were hybridized with (α-^32^P) -dATP-labeled DNA probes. Hybridization was performed in PerfectHyb plus hybridization buffer (Sigma, Germany) for 16 h at 68°C. The membranes were washed once for 10 min with 2 × SSC and 0.5% SDS, twice for 20 min with 0.5 × SSC and 0.1% SDS, once for 20 min with 0.1 × SSC and 0.1% SDS at 68°C, and then exposed to X-ray films at −80°C. DNA probes for WRKY75 were obtained from PCR amplifications using gene-specific primers.

### GUS staining and activity assay

Histochemical detection of GUS activity was performed with 5-bromo-4-chloro-3-indolyl β-D-glucuronic acid (X-gluc) as the substrate. Plant tissues were first prefixed in ice-cold 90% (v/v) acetone for 20 min, then washed three times with GUS staining buffer (without X-gluc) before incubation in X-gluc solution [1 mM X-gluc, 50 mM NaPO4, pH 7, 1 mM K_3_Fe(CN)_6_, 1 mM K_4_Fe(CN)_6_, and 0.05% Triton X-100] under vacuum for 10 min at 22 °C, then incubated overnight at 37 °C. Chlorophyll was removed using several changes of 70% ethanol, and the tissues were subsequently photographed.

For the measurements of GUS activity, leaves were homogenized in ice-cold GUS extraction buffer (50 mM phosphate buffer, pH 7.0, 10 mM EDTA, 0.1% Triton X-100, 0.1% sodium laurylsarcosine, and 10 mM β-mercaptoethanol) and microcentrifuged at 4°C. The GUS activity in the supernatant was measured using 4-methylumbelliferyl-β-D-glucuronide as substrate (Jefferson *et al.*, 1987). The standard curves were prepared with 4-methylumbelliferone.

### Construction of transgenic overexpression lines

To generate the *35S:WRKY75* and *35S:JAZ8* construct, the cDNA fragment containing the full coding sequence was excised from a cloning plasmid and sub-cloned into the same restriction sites of the *Agrobacterium* transformation vector pOCA30, in the sense orientation behind the CaMV 35S promoter. Arabidopsis transformation was performed by the floral dip procedure (Clough and Bent, 1998). Seeds were collected from the infiltrated plants and selected on half-strength Murashige and Skoog (MS) medium containing 50 μg ml^-1^ kanamycin. Kanamycin-resistant plants were transferred to soil 8 d after germination and were grown in an artificial growth chamber at 22 °C with a 16 h light/ 8 h dark photoperiod.

### Germination assays and root length measurement

Seeds were grown on half-strength MS medium with 0, 20, 50, 75 or 100 µM MeJA, chilled at 4°C for 3 d, and transferred to the growth room. Germination was scored as radicle emergence from the seed coat and endosperm. Root lengths of fifteen 14 day-old seedlings for each genotype and treatment were measured using a vernier caliper and presented. The experiments were repeated three times with similar results.

### Pathogen infection

*Botrytis cinerea* and *Alternaria brassicicola* were grown on 2 × V8 agar, as described previously (Mengiste *et al.*, 2003). To infect plants, conidia were collected from a 10 day-old culture, and the spore density was adjusted in Sabouraud maltose broth (SMB) buffer and sprayed using a Preval sprayer. Plants inoculated with a suspension of 1×10^5^ spores ml^-1^ in SMB buffer were maintained at high humidity with a transparent cover in a growth chamber, and symptom development was observed from 5 dpi (days post inoculation) to 10 dpi. Biomass of the fungal pathogen was quantified by RT–PCR of total RNA isolated from inoculated plants. For drop inoculation and GUS staining, a single 3 μl drop of a suspension of 2×10^5^ spores ml^-1^ in SMB buffer was placed on each leaf.

### Yeast two-hybrid screening and confirmation

The full-length *WRKY75*coding sequednce (CDS) was cloned into the bait vector pGBKT7 and then transformed into the yeast strain Y2HGold (Clontech, USA). Two-hybrid screening was performed via the mating protocol described in Clontech’s Matchmaker Gold Yeast Two-Hybrid user manual. To confirm protein-protein interactions, the full-length JAZ8 coding sequences (CDSs) were cloned into the prey vector pGADT7.

### Bimolecular fluorescent complementation (BiFC) assays

The cDNA sequences of enhanced YFP fragments, 173 amino acids located in the N terminus (nYFP), and 64 amino acids located in the C terminus (cYFP), were amplified by PCR and cloned into the *Xba*I-*Xho*I and *Bam*HI-*Xho*I sites of pFGC5941 to generate pFGC-nYFP and pFGC-cYFP, respectively. The full-length *WRKY75* CDS was inserted into pFGC-cYFP to generate a C-terminal in-frame fusion with cYFP, while *JAZ4* and *JAZ8* CDSs were introduced into pFGC-nYFP to form N-terminal in-frame fusions with nYFP. The resulting plasmids were introduced into *Agrobacterium tumefaciens* (strain EHA105), and infiltration of *N. benthamiana* was performed as described previously (Hu *et al.*, 2013). Infected tissues were analysed 48 h after infiltration. YFP and 49,6-diamidino-2-phenylindole fluorescence was observed under a confocal laser scanning microscope (Olympus, Japan). The primers used for BiFC are listed in Supplementary Table S2.

### Co-immunoprecipitation assays

For co-immunoprecipitation (coIP) assays, *WRKY75* and *JAZ8* were individually cloned into tagging plasmids behind the Myc or HA tag sequence. Myc-fused WRKY75 and HA-fused JAZ8 were introduced into *Agrobacterium tumefaciens* and simultaneously injected into tobacco leaves for coexpression for 48 h. Coimmunoprecipitation assays were performed using tobacco protein extracts. Briefly, HA-fused JAZ8 was immunoprecipitated using an anti-HA antibody diluted 1: 5000 in 20 mM Tris-HCl, pH 7.6, supplemented with 150 mM NaCl, 0.1% Tween 20, and 5% skimmed milk powder, and the coimmunoprecipitated proteins were then detected using an anti-Myc antibody (Sigma-Aldrich, Germany).

### Chromatin immunoprecipitation assays

Chromatin immunoprecipitation (ChIP) assays were performed essentially in accordance with previously described protocols (Saleh *et al.*, 2008). Five week-old GFP-WRKY75 plants were spray inoculated with *B.cinerea* for 0 d or 2 d, and these GFP-WRKY75 plants were used for ChIP assays. The GFP antibody was used to immunoprecipitate the protein-DNA complex, and the precipitated DNA was purified using a PCR purification kit for qRT–PCR analysis. The ChIP experiments were performed three times. Chromatin precipitated without antibody was used as the negative control, while the isolated chromatin before precipitation was used as the input control. ChIP results are presented as a percentage of input DNA. The primers used for qRT–PCR amplification of different promoters are listed in Supplementary Table S2.

### Transient expression assays

The transient expression assays were performed in *N. benthamiana* leaves. The nuclear localization signal (NLS) was fused with a GFP reporter gene behind the native promoter of *ORA59*. The full-length CDSs of *JAZ8*, *GUS*, and *WRKY75* were driven by the CaMV 35S promoter. These constructs werethen introduced into *Agrobacterium tumefaciens* (strain EHA105). Infected tissues were analysed 48 h after infiltration. The GFP signal was observed under a confocal laser scanning microscope (Olympus). All experiments were repeated with five independentbiological replicates with similar results.

## Results

### Altered response to necrotrophic pathogens resulting from knock-down or ectopic expression of *WRKY75*

During the past few years, several WRKY proteins have been demonstrated to be involved in the regulation of transcriptional reprogramming associated with plant defense responses (Rushton *et al.*, 2010; Chen *et al.*, 2013a). However, the exact mechanism(s) underlying their roles in plant defense responses remain largely unknown. To further investigate the functions of Arabidopsis WRKY transcription factors in plant defense responses, we re-screened approximately 44 WRKY-associated T-DNA insertion mutants and RNAi lines to identify additional WRKY proteins that may participate in plant basal defense (Supplementary Table S3). The tolerance of these mutants to *B. cinerea* infection was first compared with that of wild-type (WT, Col) plants. Based on the obviously severe necrotic symptoms, one RNAi line of *WRKY75* (*WRKY75RNAi*) was isolated and used for further characterization.

**Fig. 1.**
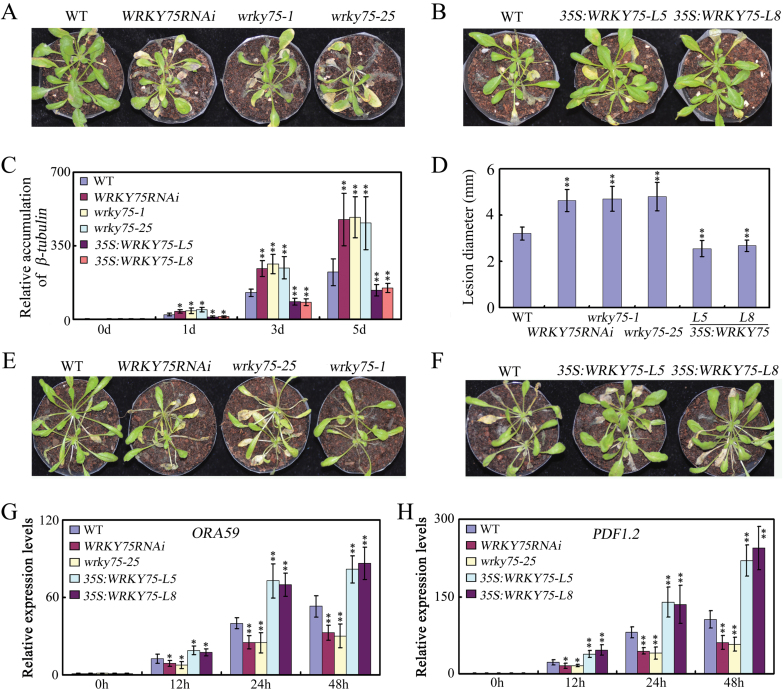
Mutation and ectopic expression of *WRKY75* result in altered responses to *B.cinerea* and *A. brassicicola.* (A). Disease symptom development. Leaves of the indicated genotypes were inoculated by spraying with a spore suspension of *B.cinerea*. Plants were maintained at high humidity and disease symptoms were photographed at 5 dpi. Scale bar=1 cm .(B). Accumulation of *B.cinerea* β-tubulin mRNA. Total RNA was isolated from inoculated plants at 1, 3 and 5dpi, and qRT–PCR was performed with *B.cinerea* β-tubulin gene-specific primers. *ACTIN2* and *UBQ5* were used as internal controls.(C) The lesion sizes on detached rosette leaves from five week-old plants at 3 dpi with *B. cinerea* spores. (D). Disease symptom development. Leaves of the indicated genotypes were inoculated by spraying with a spore suspension of *A. brassicicola*. Plants were maintained at high humidity and disease symptoms were photographed at 8 and 10 dpi. Scale bar=1 cm. (E). Disease symptom development. Leaves of the indicated genotypes were inoculated by spraying with a spore suspension of *B.cinerea*. Plants were maintained at high humidity and disease symptoms were photographed at 7 dpi. Scale bar=1 cm. ( F). Disease symptom development. Leaves of the indicated genotypes were inoculated by spraying with a spore suspension of *A. brassicicola*. Plants were maintained at high humidity and disease symptoms were photographed at 8 and 10 dpi. Scale bar=1 cm. (G, H). Expression of *ORA59* and *PDF1.2* in the indicated genotypes after inoculation with *Botrytis* for 0, 12, 24, and 48d. *ACTIN2* and *UBQ5* were used as internal controls. In C, D, G, and H, values are mean ±SE (*n*=3 experiments), and asterisks indicate significant differences compared with WT based on one way ANOVA (*P˂0.05;**P˂0.01).

To further confirm the role of *WRKY75* in defense against *B. cinerea*, we also obtained two other *wrky75* T-DNA insertion mutants, namely *wrky75-1*(SALK_101367) and *wrky75-25* (N121522; Encinas-Villarejo *et al.*, 2009; Rishmawi *et al.*, 2014; Lei *et al.*, 2014; Zhang *et al.*, 2018;Supplementary Fig.S1A,B). *WRKY75RNAi*, *wrky75-1*, *wrky75-25*, and WT seeds were germinated simultaneously and then transferred to soil. Five week-old plants were then spray inoculated with a *B. cinerea* spore suspension at a density of 5×10^4^ spores ml^-1^. Leaves showing necrotic symptoms were evaluated for disease severity. *B. cinerea* infection caused necrotic symptoms, but necrosis remained localized to specific sites in wild-type (Col-0) plants (Fig. 1A). At 5 dpi, among the total 256 wild-type leaves, only 38 leaves (about 15%) exhibited disease symptoms (Fig. 1A). In contrast, necrotic symptoms rapidly increased in severity during infection in *wrky75* mutant plants, with approximately 56% of leaves found to be severely decayed at 5 dpi (Fig. 1A). In addition, higher expression of *B. cinerea* β-tubulin mRNA occurred in *wrky75* mutant plants (Fig. 1B). Moreover, larger lesion size was observed on drop-inoculated leaves of *wrky75* mutant plants (Fig. 1C). The *wrky75* mutants were also tested for response to *A. brassicicola*, another necrotrophic fungal pathogen that causes black spot disease on cruciferous (van Wees *et al.*, 2003) plants. The *wrky75* mutants were still more sensitive to *A. brassicicola,* as represented by greater number and larger lesions, compared with WT plants (Fig. 1D; Supplementary Fig. S2). Thus, knock-down of *WRKY75* dramatically enhanced susceptibility to necrotrophic fungal pathogens.

To further characterize the role of *WRKY75* in defense against necrotrophic fungal pathogens, we compared pathogen growth in *35S:WRKY75* transgenic plants with that in WT plants. Two transgenic lines, namely *35S:WRKY75-L5* and *35S:WRKY75-L8*, which showed normal plant morphology and grew at a similar rate as WT plants, were then selected for further analysis (Supplementary Fig. S1C). In contrast to *wrky75* mutant plants, among the total of 235 leaves of *35S:WRKY75* transgenic plants, only about 24 leaves (about 10%) showed disease symptoms at 7 dpi upon *B.cinerea* infection (Fig.1E). Similarly, lower expression of *B. cinereal* β-tubulin mRNA, and smaller lesion size was observed in *35S:WRKY75* transgenic plants (Fig. 1B,C). The *35S:WRKY75* transgenic plants were also more resistant to *A. brassicicola* compared with WT plants (Fig. 1F; Supplementary Fig.S2). Constitutive overexpression of *WRKY75* thus enhanced tolerance toward necrotrophic fungal pathogens and decreased development of disease symptoms in the transgenic plants. These results confirm that WRKY75 plays an important role in plant defense against necrotrophic pathogens.

To explore the molecular basis of the altered responses of the *wrky75* mutants and *WRKY75*-overexpressing transgenic plants to the necrotrophic fungal pathogens, we characterized the expression of several defense-related genes in the JA signaling pathway in these plants after infection by *B. cinerea*. These genes included OCTADECANOID-RESPONSIVE ARABIDOPSIS (*ORA59*), and PLANT DEFENSIN gene *PDF1.2*. *ORA59* has been well characterized for its role in defense against JA-associated pathogens through directly activating *PDF1.2* expression (Pré*et al*., 2008). As shown in Fig. 1G, H, qRT–PCR analyses revealed that these defense-related genes were reduced in *wrky75* mutants, but showed enhanced expression in *WRKY75*-overexpressing plants compared with WT Arabidopsis. Taken together, these results demonstrate that the expression of defense-related genes in the JA signaling pathway was down-regulated in *wrky75* mutants and up-regulated in *WRKY75*-overexpressing plants.

### Temporal expression of *WRKY75*

**Fig. 2.**
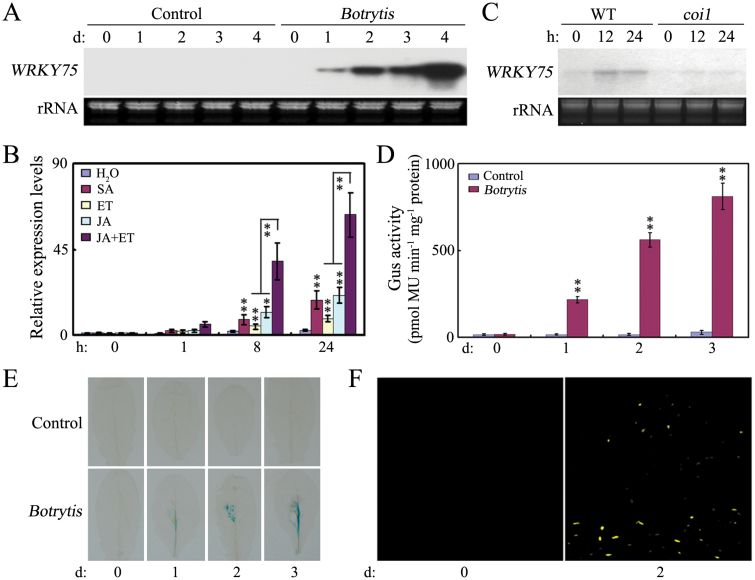
Induced expression of *WRKY75*. (A) Expression of *WRKY75* after inoculation with *B.cinerea*. Total RNA was isolated from inoculated leaves harvested at 0, 1, 2, 3, and 4 dpi and probed with a *WRKY75* fragment. Ethidium bromide–stained ribosomal RNA was used as a loading control. (B) Expression of *WRKY75* after treatment with SA, ET, JA, or combined JA and ET (time course for 0 h, 1 h, 8 h, and 24 h). RNA was extracted from four week-old Arabidopsis plants (Col-0) at given times after spraying with H_2_O, salicylic acid (SA; 2 mM), jasmonic acid (JA; 0.1mM), ethylene (ET; 0.1 mM), or combined JA and ET. *ACTIN2* and *UBQ5* were used as internal controls. (C) Expression of *WRKY75* in *coi1* mutant after treatment with JA for 12 h and 24h. RNA samples were prepared from four week-old Arabidopsis plants (WT) and *coi1* at given times after spraying with jasmonic acid (JA). Isolated RNAs were probed with a *WRKY75* fragment. Ethidium bromide–stained ribosomal RNA was used as a loading control. (D) GUS activity analysis of *WRKY75* in transgenic plants harboring *WRKY75*_*Pro*_*:GUS* after inoculation with *B.cinerea* for 0, 1, 2, and 3 d, respectively. (E) Images of samples of transgenic plants analysed for GUS activity in (D). Scale bar=0.5 cm. (F) YFP detection of WRKY75 in *wrky75-25* mutant background that harbors the *WRKY75:YFP-WRKY75:3’-WRKY75* construct. YFP signal was determined using leaves of these plants that were inoculated with *Botrytis* for 0 d and 2 d. In B, and D, values are mean ±SE (*n*=3 experiments), and asterisks indicate significant differences compared with controls based on one way ANOVA (**P<0.01).

WRKY75 appears to act as a positive regulator in plant basal defense against necrotrophic fungal pathogens. Northern blotting, detection of β-glucuronidase (GUS) activity, and GFP fluorescence were used to examine the inducibility and temporal kinetics of *WRKY75* expression during infection. As shown in Fig. 2A–C, *WRKY75* expression was strongly induced by *B. cinerea* infection, slightly induced by SA, ET and JA, with enhanced induction upon combined treatment of ET and JA. Moreover, induced expression of *WRKY75* was partially dependent on CORONATINE INSENSITIVE1 (COI1) that forms a functional E3 ubiquitin ligase SCF^COI1^. Consistent with the northern blot analysis, GUS staining further confirmed the induced expression of *WRKY75* by *B. cinerea* infection (Fig. 2D, E). To further understand *WRKY75* expression patterns, the accumulation of WRKY75 protein upon *B. cinerea* infection was also determined by observation of yellow fluorescent protein (YFP) fluorescence in leaves of *WRKY75:YFP-WRKY75:3′-WRKY75* transgenic plants inoculated with *B. cinerea*. No YFP signal was observed before treatment, while strong YFP signals were observed in *B. cinerea-*infected leaves (Fig. 2F). Taken together, these results indicate that WRKY75 may be involved in a plant basal defense response against necrotrophic fungal pathogens.

### WRKY75 acts upstream of *ORA59* and directly regulates its expression

**Fig. 3.**
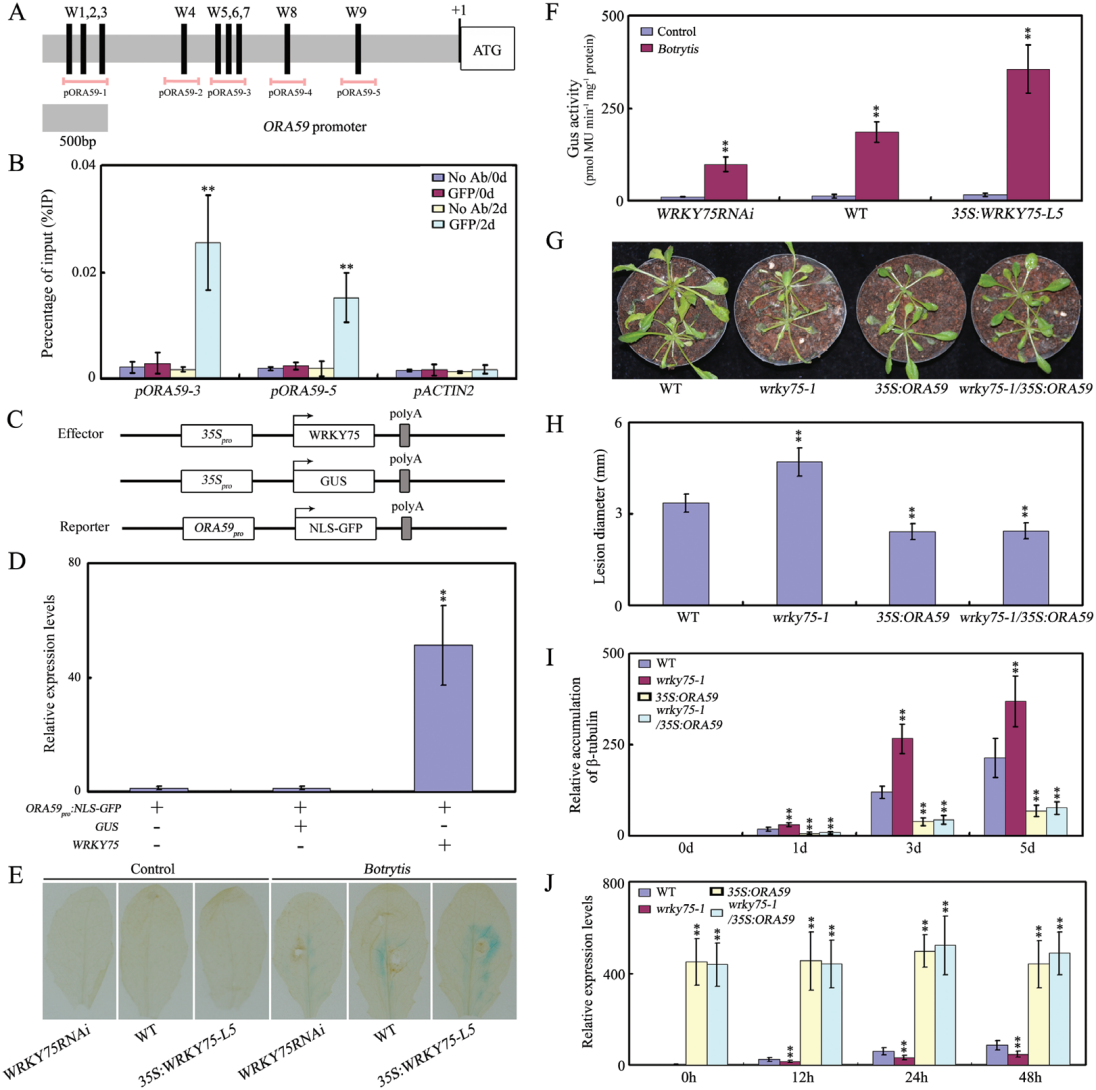
*ORA59* is a direct target of WRKY75. (A) The promoter structure of the *ORA59* gene and fragment used in the ChIP assay. The upper panel shows schematic representation of the *ORA59* promoter regions containing W-box clusters. Only perfect W-boxes (T/CTGACC/T, black bar) are depicted. The diagram indicates the number and relative position of the W-boxes in the respective promoters relative to the ATG start codon. In the promoter fragment names, the prefix p indicates promoter. Pink lines indicate the sequences detected by ChIP assays. (B) ChIP assays were performed with chromatin prepared from *WRKY75:YFP-WRKY75:3’-WRKY75* transgenic plants infected with *B.cinerea* for 0 d and 2 d.Using an anti-GFP antibodyChIP results are presented as a percentage of input DNA. (C) Schematic of the *ORA59:NLS-GFP* reporter and *WRKY75* and *GUS* effectors. (D) qRT–PCR analysis of the accumulation of *GFP* transcripts. Total RNAs were extracted from leaves of *N. benthamiana* co-infiltrated with combinations of various constructs in (C). (E, F) GUS activity analysis of *ORA59* in various *WRKY75* genetic backgrounds harboring *ORA59:GUS* after inoculation with *Botrytis* for 0 d and 1 d. The *ORA59:GUS* reporter was inhibited in *WRKY75RNAi* plants while it was activated in *35S:WRKY75-L5* plants when compared with that in the WT. (G) Disease symptom development. Leaves of the indicated genotypes were inoculated by spraying with a spore suspension of *B.cinerea*. Plants were maintained at high humidity and disease symptoms were photographed at 5 dpi. (H) The lesion sizes on detached rosette leaves from five week-old plants at 3 dpi with *B. cinerea* spores. (I) Accumulation of *B.cinerea* β-tubulin mRNA. Total RNA was isolated from inoculated plants at 4 dpi and qRT–PCR was performed with *B.cinerea* β-tubulin gene-specific primers. *ACTIN2* and *UBQ5* were used as internal controls. (J) Expression of *PDF1.2* in the indicated genotypes after inoculation with *B.cinerea* for 0, 12, 24, and 48 h. *ACTIN2* and *UBQ5* were used as internal controls. In B, D, F, H-J, values are mean ±SE (*n*=3 experiments), and asterisks indicate significant differences compared with controls based on one way ANOVA (**P<0.01).

WRKY transcription factors function by binding directly to a putative *cis*-element in their target gene promoters, the W-box (T/CTGACC/T; Eulgem *et al.*, 2000; Ulker and Somssich, 2004). Our data suggest that WRKY75 may play an important role during plant-pathogen interactions by positively modulating the expression of defense-related genes in the JA signaling pathway. Interestingly, a search of the Arabidopsis genome uncovered several putative W-box elements in the promoter of the JA signaling-associated gene *ORA59*. The presence of these elements indicates that the observed modulation may be caused by direct interaction with WRKY factors, including WRKY75. To examine whether WRKY75 can directly regulate *ORA59* expression, we first conducted chromatin immunoprecipitation (ChIP) experiments using *WRKY75:YFP-WRKY75:3’-WRKY75* transgenic plants (Rishmawi *et al.*, 2014). These experiments showed that WRKY75 directly interacted with the *ORA59* promoter when tested with the primer combinations encompassing either W5-7 or W9 upon *B. cinereal* infection (Fig.3A, B; Supplementary Table S2), suggesting that WRKY75 directly regulates *ORA59* transcription.

To further confirm the positive regulatory function of WRKY75, we also performed transient expression assays of *Nicotiana benthamiana* leaves. The *ORA59:NLS-GFP* reporter was used as a reporter plasmid. Effector plasmids were generated that contained either a *WRKY75* or *GUS* gene driven by the cauliflower mosaic virus (CaMV) 35S promoter (*35S:WRKY75* and *35S:GUS*; Fig. 3C). As shown in Fig. 3D, coexpression of the *WRKY75* gene resulted in enhanced GFP expression compared with the control. This supports the hypothesis that WRKY75 is a positive regulator of JA-mediated defense signaling.

We then examined the expression of *ORA59:GUS* reporter in different *WRKY75* transgenic lines after incubation with *B.cinerea*. In *WRKY75RNAi* background, *ORA59:GUS* reporter was obviously inhibited compared with that in the WT (Fig. 3E, F). In contrast to this, in the *35S:WRKY75-L5* background, *ORA59:GUS* expression was greatly enhanced compared with that in the WT (Fig. 3E, F). Thus, the GUS activity analysis agrees with the binding between WRKY75 and the W-boxes in the promoter of *ORA59* (Fig. 3B).

The phenotypic analysis, biochemical and molecular data demonstrated that the transcription factor WRKY75 positively regulates plant defense against necrotrophic pathogens through the direct activation of *ORA59* expression. To further confirm this conclusion, the genetic relationship between *WRKY75* and *ORA59* was explored. The *wrky75-1* mutant was crossed with *35S:ORA59* transgenic plants, and the disease symptoms of *wrky75-1* and *wrky75-1*/*35S:ORA59* were examined. Under our experimental conditions, we detected an obviously enhanced tolerance in *35S:ORA59* plants, and mutation of *WRKY75* did not change this in terms of accumulation of *B. cinereal* β-tubulin, lesion size, and *PDF1.2* expression (Fig. 3G–J), although the *wrky75-1* mutant showed enhanced susceptibility to the necrotrophic fungal pathogen (Fig. 3G–J). Thus, the genetic analysis indicated that WRKY75 acts upstream of *ORA59* to positively regulate plant defense against necrotrophic pathogens.

### Physical interaction of WRKY75 with JAZ proteins

**Fig. 4.**
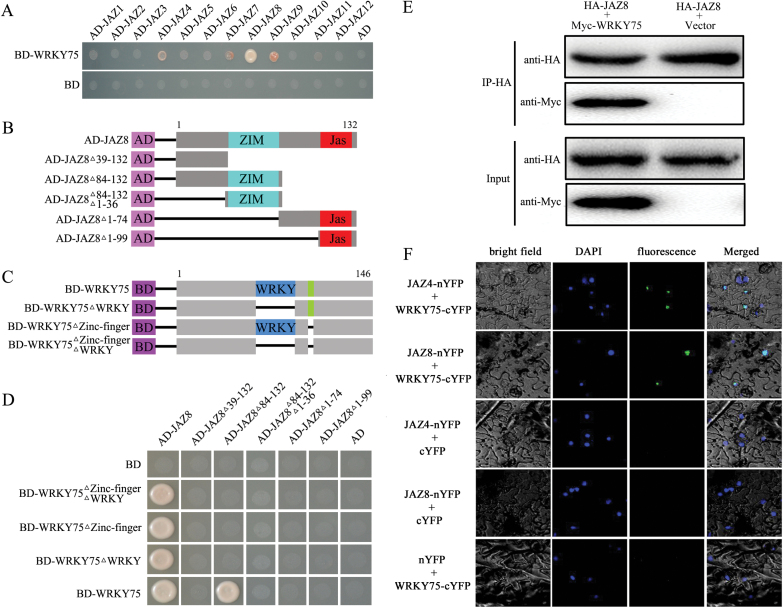
Interactions between JAZ repressors and WRKY75. (A) Yeast two-hybrid assay analysis. Interaction was indicated by the ability of cells to grow on synthetic dropout medium lacking Leu, Trp, His, and Ade. The GAL4 activation domain expressed by pGADT7 (shown as AD) was used as negative controls. (B) Diagram of full-length and truncated JAZ8 constructs. (C) Diagram of full-length and mutated WRKY75 constructs. (D) The N-terminusof JAZ8 (containing the ZIM domain) is responsible for interaction of JAZ8 with WRKY75, and both the WRKY domain and zinc-finger domain are important for the interaction between JAZ8 and WRKY75. Interactions were indicated by the ability of yeast cells to grow on synthetic dropout medium lacking Leu, Trp, His, and Ade. The empty pGADT7 prey vector and pGBKT7 bait vector was used as negative controls. (E) Co-IP analysis. HA-fused JAZs were immunoprecipitated using anti-HA antibody, and co-immunoprecipitated Myc-WRKY75 was then detected using anti-Myc antibody. Protein input for HA-JAZs and Myc-WRKY75 in immunoprecipitated complexes were also detected and are shown. (F) BiFC analysis. Fluorescence was observed in nuclear compartments of *N. benthamiana* leaf epidermal cells; the fluorescence resulted from complementation of the N-terminal portion of YFP fused to JAZ factors (JAZ-nYFP) with the C-terminal portion of YFP fused to WRKY75 (WRKY75-cYFP). No signal was observed from negative controls. DAPI, 49,6-diamidino-2-phenylindole.

To understand how WRKY75 participates in plant basal defense against necrotrophic pathogen infection, we used the yeast two-hybrid system to identify its potential interaction partners. The full-length coding sequence of *WRKY75* was fused to the Gal4 DNA binding domain of the bait vector (BD-WRKY75). Yeast cells harboring the bait were transformed with a cDNA library containing inserts for prey proteins fused to GAL4-AD. After screening, three independent clones encoding JAZ8 were identified by prototrophy for His and Ade. To confirm the interaction of these clones in yeast, their open reading frame sequences were fused with the AD domain of the pGADT7 vector and used for further interaction experiments with WRKY75. The bait and prey vectors were co-transformed into yeast, and protein–protein interactions were tested (Fig. 4A). We investigated interactions of WRKY75 with all 12 Arabidopsis JAZ proteins in the yeast two-hybrid system. Besides JAZ8, WRKY75 also slightly interacted with JAZ4, JAZ7, and JAZ9 (Fig. 4A).

To investigate which region of JAZ8 is required for interaction with WRKY75, we fused five truncated JAZ8 variants to the AD domain of the pGADT7 vector (Fig. 4B). The interaction between these derivatives and WRKY75 was then assayed using the yeast two-hybrid system. The data revealed that the 83 N-terminal residues of JAZ8 (containing the ZIM domain) were specifically responsible for the interaction (Fig. 4C). This result indicates that the N-terminal fragment including the ZIM domain of JAZ8 is necessary for its interaction with WRKY75.

To identify the WRKY75 region responsible for the WRKY75-JAZ8 interaction, we performed an additional directed yeast two-hybrid analysis using pGBKT7 vectors containing a WRKY domain mutant, a zinc finger domain mutant, or both (Fig. 4D). WRKY75 proteins containing either or both of these mutations were still able to interact with the full-length JAZ8 protein but not with the 83 N-terminal JAZ8 residues (Fig. 4C). These results demonstrate that WRKY and zinc finger domains, while not critical, are still important elements in the interaction between WRKY75 and JAZ8.

Interactions of WRKY75 with JAZ proteins were further corroborated by coIP assays and bimolecular fluorescence complementation (BiFC). JAZ4 and JAZ8 were used as representatives in the coIP and BiFC assays. For the coIP analysis, Myc-WRKY75 and HA-JAZ8 were co-expressed in *N. benthamiana* leaves. The protein complexes were incubated with anti-HA and A/G-agarose beads and then separated using SDS-PAGE for immunoblotting with anti-Myc antibody. As shown in Fig. 4E, the WRKY75 proteins could be pulled down by JAZ8. 

To determine whether these interactions also occur in plant cells, we then used BiFC analysis. Full-length JAZ4 and JAZ8 proteins and WRK75 were fused to the N-terminal region of a YFP fragment, yielding JAZ-nYFP and WRKY75-cYFP, respectively. *Agrobacterium* cells harboring the corresponding interaction pair were infiltrated into *N. benthamiana* leaves. In parallel, empty vectors in combination with each fusion construct were also co-infiltrated into *N. benthamiana* leaves, as controls. After 48h incubation, YFP signals were observed with fluorescence microscopy. The samples co-infiltrated with an interaction pair showed YFP fluorescence in the cell nuclei, whereas none of the control samples yielded any signal (Fig. 4F). These results indicate that WRKY75 and its partners co-localize and interact in plant cell nuclei. Taken together, these results demonstrate that WRKY75 physically interacts with JAZ proteins.

### JAZ8 represses transcriptional function of WRKY75

**Fig. 5.**
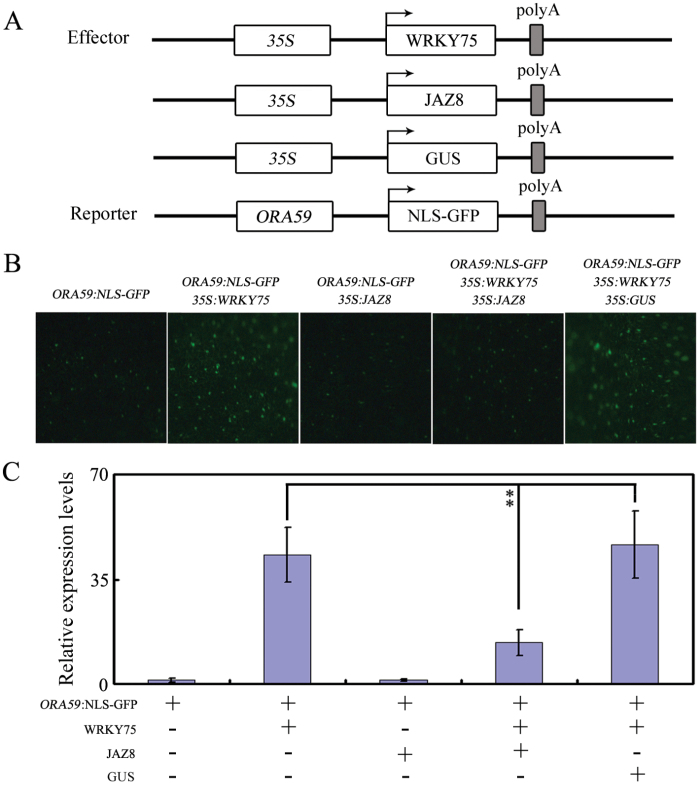
JAZ8 represses WRKY75 transcriptional function. (A) Schematic representation of the *ORA59:NLS-GFP* reporter and *WRKY75*, *JAZ8,* and *GUS* effectors. (B) Transient expression assays showed that JAZ8 represses transcriptional activation of WRKY75. GFP fluorescence was detected 48 h after co-infiltration with the indicated constructs. The experiment was repeated three times with similar results. Scale bar=50 μm. (C) qRT–PCR analysis of the accumulation of *GFP* transcripts. Total RNA was extracted from leaves of *N. benthamiana* co-infiltrated with combinations of various constructs in (A) *EF1α* was used as an internal control. Values are mean ±SE (*n*=3 experiments), and asterisks indicatesignificant differences compared with controls based on one way ANOVA (**P<0.01).

Because JAZ proteins directly interact with WRKY75, we hypothesized that these physical interactions might interfere with the function of WRKY75 as a transcription factor. To test this possibility, *35S:WRKY75*, *35S:JAZ8*, and *35S:GUS* were used as effector plasmids and *ORA59:NLS-GFP* was again used as a reporter plasmid (Fig. 5A). When the reporter construct was transformed into *N. benthamiana* leaves and kept at 22°C, a relatively low fluorescence signal was observed (Fig. 5B). When *ORA59:NLS-GFP* was co-infiltrated into *N. benthamiana* leaves along with *35S:WRKY75*, much stronger fluorescence signals were observed (Fig. 5B). In contrast, co-infiltration of *ORA59:NLS-GFP* with *35S:JAZ8* generated relatively lower fluorescence signals (Fig. 5B). In addition, co-infiltration of *ORA59:NLS-GFP* with *35S:JAZ8* and *35S:WRKY75* also generated dramatically weaker fluorescence signals in comparison with co-infiltration of *ORA59:NLS-GFP* with *35S:WRKY75* (Fig. 5B). As a control, co-infiltration of *ORA59:NLS-GFP* with *35S:GUS* and *35S:WRKY75* was performed, but no obvious differences in fluorescence signals were observed compared with co-infiltration of *ORA59:NLS-GFP* and *35S:WRKY75* (Fig. 5B). Taken together, these results demonstrate that the JAZ8 protein represses the transcriptional function of WRKY75.

To further verify the effect of JAZ8 on WRKY75 transcriptional function, we analyzed relative *GFP* expression in *N. benthamiana* leaves. As shown in Fig. 5C, we detected high expression of *GFP*in *ORA59:NLS-GFP*- and *35S:WRKY75*-co-infiltrated *N. benthamiana* leaves. In contrast, coexpression of JAZ8 protein with WRKY75 suppressed *GFP* transcript accumulation (Fig. 5C). These results further support the notion that JAZ proteins repress the transcriptional function of WRKY75.

### Repression of disease resistance by overexpression of *JAZ8*

**Fig. 6.**
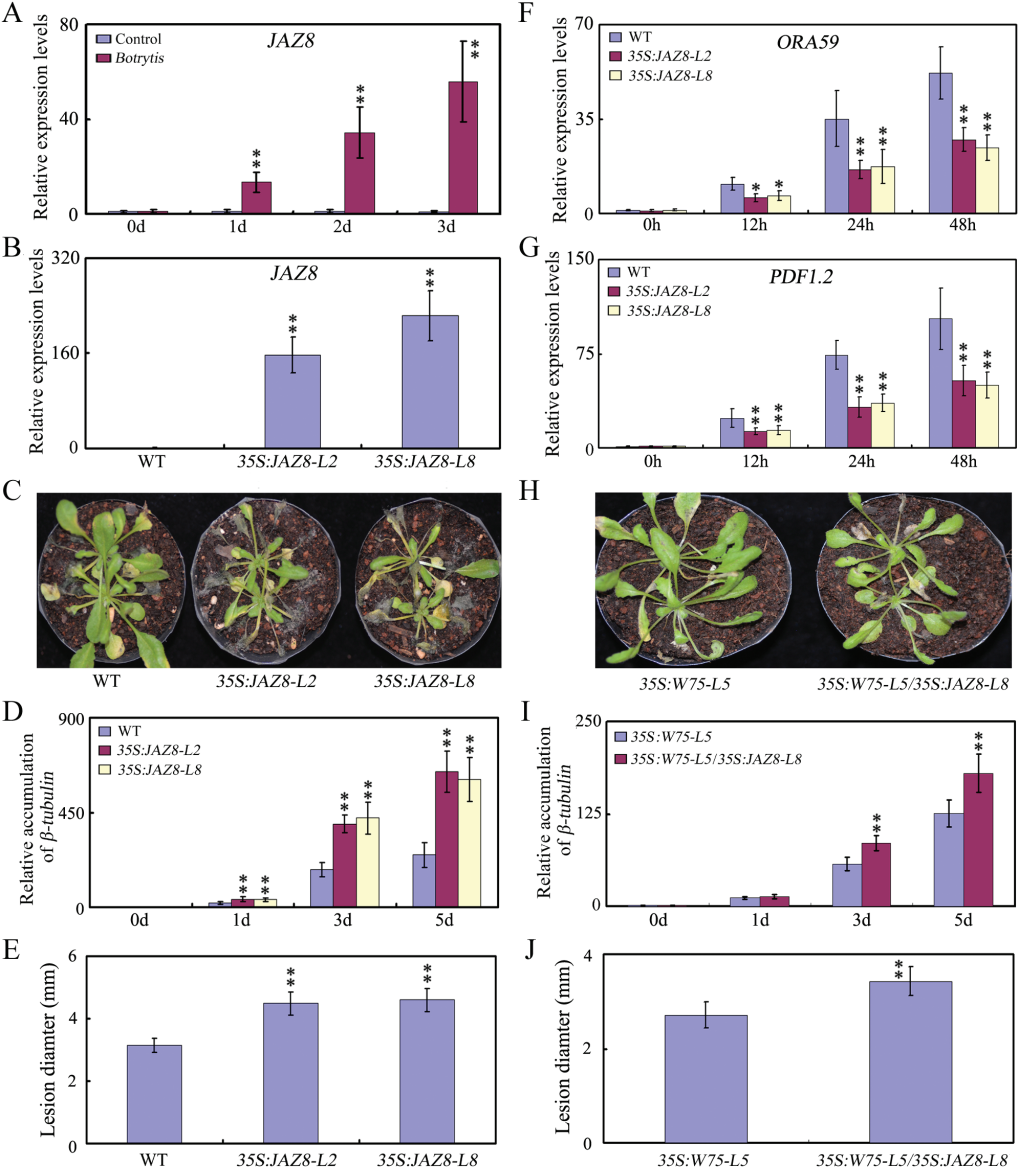
Phenotypic characterization of the *JAZ8* overexpression plants upon *B.cinerea* infection. (A) qRT–PCR analysis of *JAZ8* transcripts in leaves after inoculation with *B.cinerea* for 0, 1, 2, and 3 d. (B) qRT–PCR analysis of *JAZ8* transcripts in *JAZ8* overexpression lines. (C) Disease symptom development. Leaves of WT and *JAZ8* overexpression plants were inoculated by spraying with a spore suspension of *B.cinerea*. Plants were maintained at high humidity and disease symptoms were photographed at 6 dpi. (D) Accumulation of *B.cinerea β-tubulin*. Total RNA was isolated from inoculated WT and *JAZ8* overexpression plants at 0, 1, 3 and 4 dpi and qRT-PCR was performed with *B.cinerea β-tubulin* gene-specific primers. (E) The lesion sizes on detached rosette leaves from 5-week-old WT and *JAZ8* overexpression plants at 3 days post-inoculation with *B. cinerea* spores. (F, G) Expression of *ORA59* and *PDF1.2*. Expression of *ORA59* and *PDF1.2* in WT and *JAZ8* over-expression lines after inoculation with *Botrytis* for 0, 12, 24, and 48 h, respectively. *ACTIN2* and *UBQ5* were used as internal controls. (H) Disease symptom development. Leaves of *35S:WRKY75-L5* and *35S:WRKY75-L5/35S:JAZ8-L8* plants were inoculated by spraying with a spore suspension of *B.cinerea*. Plants were maintained at high humidity and disease symptoms were photographed at 6 dpi. (I) Accumulation of *B.cinerea β-tubulin*. Total RNA was isolated from *35S:WRKY75-L5* and *35S:WRKY75-L5/35S:JAZ8-L8* plants at 0, 1, 3 and 4 dpi and qRT-PCR was performed with *B.cinerea β-tubulin* gene-specific primers. (J) The lesion sizes on detached rosette leaves from 5-week-old *35S:WRKY75-L5* and *35S:WRKY75-L5/35S:JAZ8-L8* plants at 3 days post-inoculation with *B. cinerea* spores. In A, B, D-G, I and J, values are mean ±SE (*n*=3 experiments), and asterisks indicatesignificant differences as compared to controls based on one way ANOVA (**P<0.01).

Because several JAZ repressors interact with WRKY75 and modulate its transcriptional function, we investigated whether disruption or overexpression of the JAZ8 protein affects Arabidopsis disease response againstnecrotrophic fungal pathogens. We first found that *JAZ8* expression was strongly induced by *B. cinerea* infection (Fig. 6A). We then analysed the performance of *jaz8* mutant plants in response to *B. cinerea* infection. The *jaz8* mutants exhibited disease resistance similar to that of WT plants upon *B. cinerea* infection (Supplementary Fig. S3). *JAZ8* overexpression, however, rendered the transgenic plants (*35S:JAZ8*) more sensitive to *B. cinerea* infection (Fig. 6B–E). Consistent with this observation, transcripts of *ORA59* and *PDF1.2* were dramatically reduced in *B. cinerea*-infected transgenic 3*5S:JAZ8* plants (Fig. 6F, G). These results indicate that overexpression of *JAZ8* represses the JA signaling pathway and disease resistance response in Arabidopsis.

To further corroborate the regulatory effect of JAZ8 on the transcriptional function of WRKY75 in Arabidopsis, we investigated whether overexpression of *JAZ8* could repress the defense resistance phenotype of *WRKY75*-overexpressing plants. As shown in Fig. 6H–J, based on the larger lesion size and higher expression of β-tubulin in *33S:W75-L5/35S:JAZ8-L8*, transgenic expression of *JAZ8* was able to partially repress the phenotype of *WRKY75*-overexpressing plants in defense response to *B.cinerea* infection. These observations further support the idea that JAZ8 protein represses transcriptional function of WRKY75 in Arabidopsis.

### Knock-down or ectopic expression of *WRKY75* results in opposite responsiveness to methyl jasmonate

**Fig. 7.**
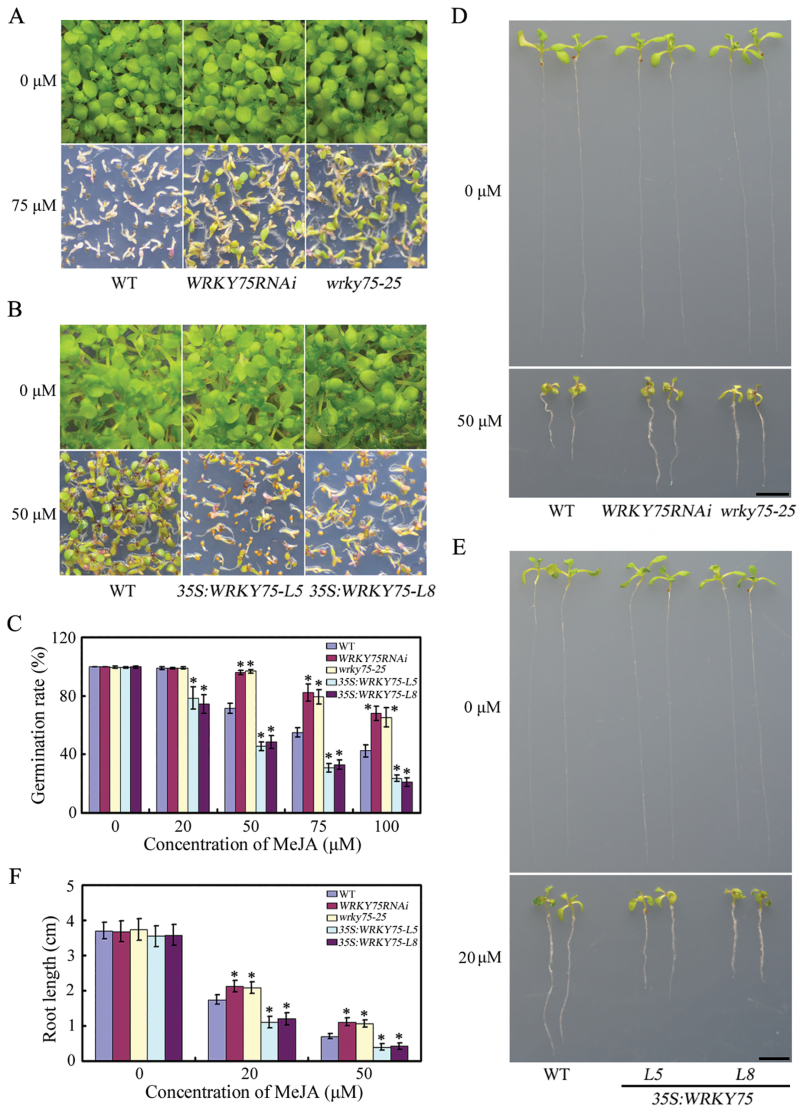
Knock-down or ectopic expression of *WRKY75* results in contrasting responses to MeJA. (A, B) Photographs of WT, *WRKY75RNAi*, *wrky75-25*(A), *35S:WRKY75-L5* and *35S:WRKY75-L8.* (B) on half-strength MS medium with the indicated concentrations (µM) of MeJA at 10 d after stratification. Scale bar=0.1 cm. (C) Germination frequency of WT, *wrky75* mutants and *WRKY75* over-expression lines were scored 6 d after stratification on various concentrations of MeJA. (D, E) Photographs of 14 day-old WT, *wrky75* mutants and *WRKY75* over-expressing seedlings grown on half-strength MS medium supplied with indicated concentrations (µM) of MeJA. Scale bar=0.5 cm. (F) Root lengths of WT, *wrky75*mutants and *WRKY75* over-expressing seedlings grown on half-strength MS medium supplied with indicated concentrations (µM) of MeJA. In C and F, values are mean ±SE (*n*=3 experiments), and asterisks indicate significant differences compared with WT based on one way ANOVA (*P<0.05).

Having demonstrated that WRKY75positively regulates jasmonate-mediated plant defense to necrotrophic fungal pathogens, we further investigated whether knock-down or ectopic expression of *WRKY75* would lead to altered responsiveness to MeJA. Previous studies have demonstrated that JA/MeJA is capable of inhibiting seed germination in *Brassica napus*, *Linum usitatissimum*, *Solanum lycopersicum*, and Arabidopsis (Wilen *et al.*, 1991; Miersch *et al.*, 2008; Oh *et al.*, 2009; Dave *et al.*, 2011). Thus we first characterized the role of *WRKY75* in seed germination. The *wrky75* mutants were less sensitive than the WT to inhibition of seed germination by JA (Fig. 7A–C). In contrast, over-expressing *WRKY75* transgenic plants were more sensitive to JA-inhibited seed germination. We also tested whether *WRKY75* plays a role in JA inhibition of root growth. As expected, knock-down or ectopic expression of *WRKY75* resulted in opposite responsiveness to MeJA in root growth compared with the WT (Fig. 7D–F). Taken together, knock-down or ectopic expression of *WRKY75* results in opposite responsiveness to MeJA, demonstrating that WRKY75 functions as an important positive regulator of JA responses.

## Discussion

Although previous studies have provided evidence that WRKY transcription factors are important components of plant defense responses, the biological roles of specific WRKY proteins in these processes are largely unknown. Considering the size of the *WRKY* gene family, functional elucidation of specific WRKY proteins under various stresses will continue to be a major challenge. In this study, we focused on the function of the Arabidopsis *WRKY75* gene in plant defense responses and disease resistance against necrotrophic fungal pathogens.

### WRKY75 acts as a positive regulator in jasmonate-mediated plant defense

Plants have evolved various adaptive mechanisms, such as defense responses towards attack from by various pathogens, to enable rapid adjustment to a continually changing environment. Among the components involved in these processes, transcriptional regulatory networks play an important role. As a class of specific transcription factors, *WRKY* genes have been demonstrated to be involved in diverse aspects of plant growth and development, and responses to biotic and abiotic stresses (Rushton *et al.*, 2010; Parinita *et al.*, 2011; Chen *et al.*, 2012; Chen *et al.*, 2017). Several WRKY members in Arabidopsis, including WRKY3, WRKY4, WRKY8, WRKY18, WRKY33, WRKY40, and WRKY60, function as positive regulators in defense againstnecrotrophic fungal pathogens (Xu *et al.*, 2006; Zheng *et al.*, 2006;Lai *et al.*, 2008; Lai *et al.*, 2011a; Chen *et al.*, 2010). In the present study, we found that *WRKY75* was strongly induced by *B. cinerea* infection at both mRNA and protein levels, and was also induced by exogenous MeJA application (Fig.2). As measured by enhanced disease symptoms and increased pathogen growth in inoculated plants, both *WRKY75 RNAi* and T-DNA insertion alleles were found to exhibit increased susceptibility to the necrotrophic fungal pathogens *B. cinerea* and *A. brassicicola* (Fig. 1). In contrast, transgenic plants constitutively expressing the *WRKY75* gene were more resistant to these necrotrophic pathogens than were WT plants (Fig. 1). Furthermore, *WRKY75* expression in the *coi1* mutant was significantly lower after MeJA treatment than in the WT, suggesting that the induced expression of *WRKY75* by JA is partially dependent on *COI1* function (Fig. 2C). These results indicate that WRKY75 positively regulates JA-mediated plant defense to necrotrophic fungal pathogens.

Resistance to necrotrophic pathogens in Arabidopsis depends on JA and ET signaling pathways, as mutations that block JA or ET signaling, such as *coi1* and *jar1* for JA, and *ein2* for ET, result in enhanced susceptibility (Glazebrook, 2005). In addition to partial COI1-dependent expression, we also observed that *wrky75* mutants after *B. cinerea* infection showed reduced expression of several defense-related genes in the JA/ET signaling pathway, including *ORA59* and *PDF1.2* (Fig. 1G, H). These results suggest that JA/ET-mediated responses that are important for defense against *B. cinerea* might be compromised in the *wrky75* mutants. Consequently, the important role of *WRKY75* in plant defense against necrotrophic pathogens may occur through its action as a positive regulator in JA/ET-mediated signaling pathways.

### Mechanisms underlying the role of WRKY75 in defense against necrotrophic pathogens

Despite their functional diversity, WRKY proteins regulate temporal and spatial gene expression primarily by binding to W-box elements of target gene promoters having the minimal consensus W-box sequence T/CTGACC/T (Eulgem *et al.*, 2000; Ulker and Somssich, 2004). The differential expression of *WRKY* genes under various environmental conditions and the transcriptional-inducing or -repressing activity of their corresponding proteins, may enable their specific roles. Identification of additional components directly regulated by WRKYs may help further elucidate the biological functions of WRKY transcription factors and their possible signaling pathways. As shown in Fig. 2, *WRKY75* was strongly induced by *B. cinerea*. Thus, the WRKY75 protein may accumulate upon *B. cinerea* infection and mediate transcriptional activation or repression of potential target genes. According to our ChIP results, WRKY75 binds to W-box elements upstream of the *ORA59* promoter during infection (Fig. 3B), indicating that *ORA59* is a direct target of WRKY75. The opposite expression patterns of *WRKY75* and *ORA59* in *WRKY75* knock-down plants and overexpression lines (Fig. 1G;Fig. 3E, F), and the up-regulation of *GFP* expression in transient expression assays (Fig. 5), further suggest that WRKY75 positively regulates *ORA59*. Furthermore, genetic analysis showed that WRKY75 functions as a positive regulator in plant defense against necrotrophic pathogens in an *ORA59*-dependent manner (Fig. 3G–J). On the basis of our results, WRKY75 therefore participates in plant defense responses against necrotrophs through the JA signaling pathway.

The plant hormone JA, ubiquitous in the plant kingdom, is required for regulation of multiple physiological processes. Previous studies have shown that JAZ proteins are key regulators of the JA signaling pathway (Kazan and Manners, 2012). As is well known, JAZ proteins block the activity of transcriptional regulators of JA responses by physically interacting with various transcription factors in resting cells. Upon perception of bioactive JAs, however, JAZ proteins are rapidly recruited by SCF^COI1^ for ubiquitination and subsequent degradation. Degradation of these JAZ proteins would activate their downstream transcription factors, resulting in the activation of downstream JA responses (Pauwels and Goossens, 2011). In Arabidopsis, several transcription factors have been shown to be targets of JAZs to positively or negatively regulate plant defense responses. For example, ETHYLENE INSENSITIVE3 (EIN3) and its closest homolog EIN3-LIKE 1 (EIL1) were recently identified as direct targets of JAZ proteins to positively mediate plant defense responses to necrotrophic fungal pathogens (Zhu *et al.* 2011). In contrast, several bHLH transcription factors (including MYC2, bHLH3, bHLH13, bHLH14 and bHLH17) interact with JAZ proteins to negatively regulate plant defense responses against *B.cinerea* (Fernández-Calvo *et al.*, 2011; Song *et al.*, 2013). In this study, we demonstrated that JAZ-targeted WRKY75 positively modulates JA-mediated plant defense by directly regulating JA-responsive genes such as *ORA59* (Figs. 3, 4).

**Fig. 8.**
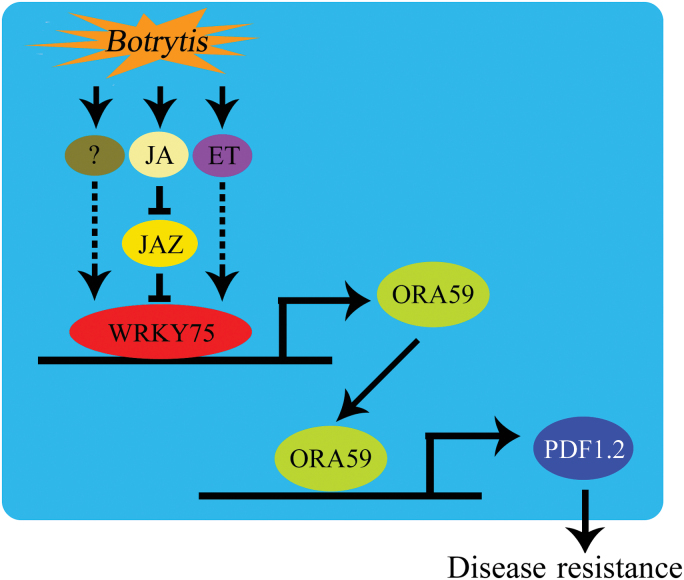
Model for WRKY75-regulated defense responses in Arabidopsis. Upon *B.cinerea* infection, production of endogenous jasmonate is induced and induces the degradation of JAZ proteins and subsequently releases WRKY75 to activate *ORA59* gene expression and downstream defense-related genes (such as *PDF1.2*). Furthermore, WRKY75 may also interact with components of ET signaling or other defense associated proteins to modulate defense response against necrotrophic pathogens.

We propose a working model illustrated in Fig. 8 to explain the molecular mechanism of *WRKY75*-regulated defense responses in Arabidopsis. Under normal growth conditions, JAZ repressors physically interact with the WRKY75 transcription factor (Fig. 4) and inhibit its transcriptional function (Fig. 5), thereby repressing expression of downstream defense-responsive genes. Upon *B. cinerea* infection, however, JAZ proteins are degraded via the SCF^COI1^-26S proteasome pathway and then WRKY75 is released to regulate its target genes (such as *ORA59*); this process may further modulate expression of JA-responsive genes essential for various JA responses (Fig. 8). Furthermore, besides interacting with JAZ repressors of JA signaling, WRKY75 may also form a complex with components of ET signaling or other defense-associated proteins, finally modulating the defense response against necrotrophic pathogens. However, it remains to be investigated how WRKY75 is regulated at both transcriptional and translational levels upon pathogen infection, and whether this mechanism is conserved for other defense-associated WRKY transcription factors. Being sessile, plants have had to develop sophisticated systems to adapt to continuously changing environments. In future, it will be interesting to investigate whether the WRKY–JAZ module also operates in cereal plants upon defense against diverse pathogens. The identification of additional defense-related genes like *WRKY75* will add to our understanding of the complex phenomenon of plant-pathogen interactions.

## Supplementary data

The following supplementary data are available at *JXB* online.

Fig. S1. qRT–PCR analysis of *WRKY75* transcripts in *WRKY75* transgenic plants.

Fig. S2. The lesion sizes on detached rosette leaves of five week-old *WRKY75* mutants and overexpression lines at 4 d post-inoculation with *A. brassicicola* spores.

Fig. S3. Phenotypic characterization of the *jaz8* mutant plants upon *B.cinerea* infection.

Table S1. A checklist outlining the RNA to qRT–PCR quality/methodology.

Table S2. Primers used in this study.

Table S3. *WRKY* genes screened in this study.

eraa529_suppl_Supplementary_File001

## Data Availability

Sequence data from this article can be found in the GenBank/EMBL libraries (https://www.ncbi.nlm.nih.gov/) under the following accession numbers: *WRKY75*(AT5G13080), *ORA59*(At1G06160), *PDF1.2*(At5G44420), *JAZ1*(AT1G19180), *JAZ2*(AT1G74950), *JAZ3*(AT3G17860), *JAZ4* (AT1G48500), *JAZ5*(AT1G17380), *JAZ6*(AT1G72450), *JAZ7*(AT2G 34600), *JAZ8*(AT1G30135), *JAZ9*(AT1G70700), *JAZ10*(AT5G13220), *JAZ11*(AT3G43440), *JAZ12*(AT5G20900), *ACTIN2*(AT3G18780), and *UBQ5*(AT3G62250).
