# Supplementary material for: The transcription factor WRKY75 positively regulates jasmonate-mediated plant defense to necrotrophic fungal pathogens
Source: J Exp Bot. 2020 Nov 9;72(4):1473–89. doi: 10.1093/jxb/eraa529 (PMC7904156; doi:10.1093/jxb/eraa529)
Supplement: eraa529_suppl_Supplementary_File001 [file eraa529_suppl_supplementary_file001.pdf]

Supplemental data for

AtWRKY75 positively regulates jasmonate-mediated plant defense to necrotrophic fungal pathogens

Author: Ligang Chen, Liping Zhang, Shengyuan Xiang, Yanli Chen, Haiyan Zhang, and Diqiu Yu*

Supplementary figures and tables:

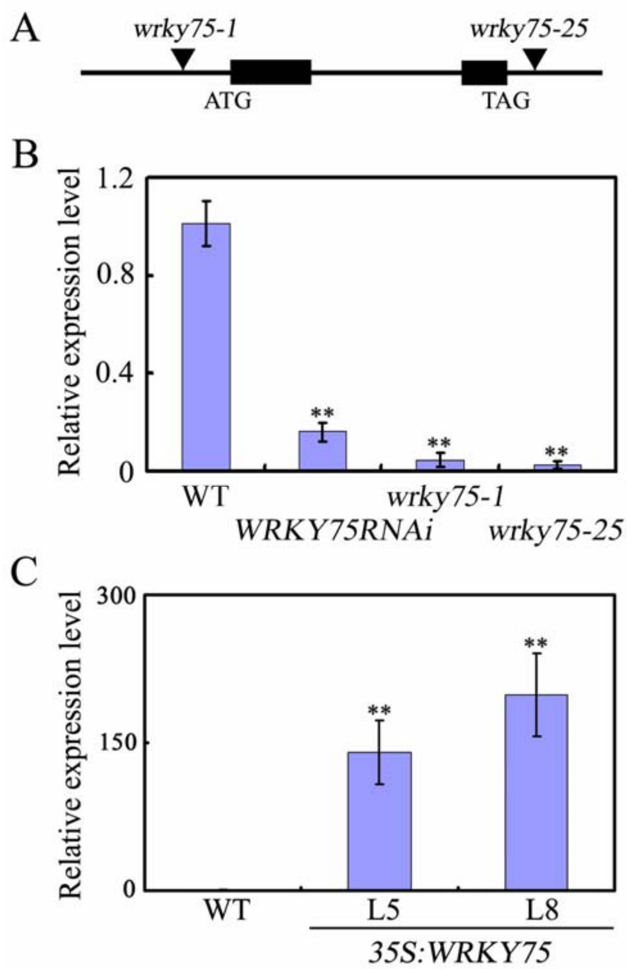

Figure S1. qRT-PCR analysis of *WRKY75* transcripts in *WRKY75* mutants and over-expression plants.

A. The T-DNA insertion position in the *wrky75* mutants.

B. qRT-PCR analysis of *wrky75* mutants. RNA was extracted from 8-day-old plants growing on 1/2 MS media. *ACTIN2* and *UBQ5* were used as internal controls. Values are mean SE (n = 3 experiments), and asterisks indicate significant differences as

compared to controls based on one way ANOVA (**P<0.01).

C. qRT-PCR analysis for *WRKY75*-overexpressing plants. RNA was extracted from 8-day-old plants growing on 1/2 MS media. *ACTIN2* and *UBQ5* were used as internal controls. Values are mean SE (n = 3 experiments), and asterisks indicate significant differences as compared to controls based on one way ANOVA (**P<0.01).

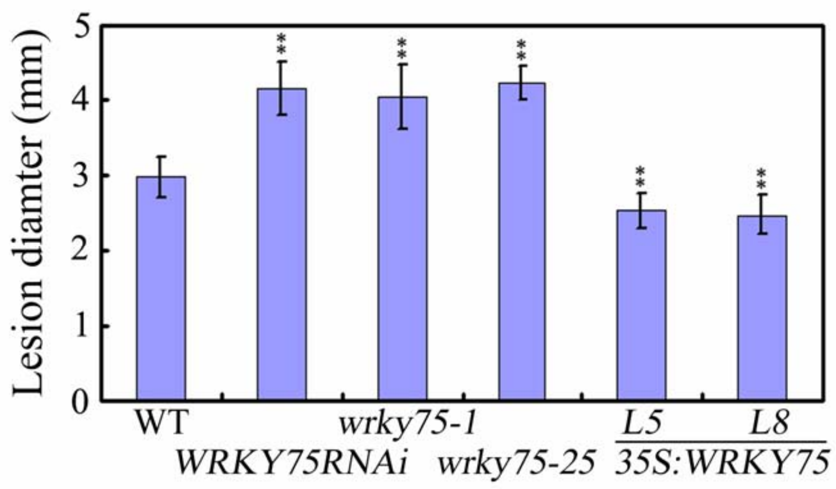

Figure S2. The lesion sizes on detached rosette leaves of 5-week-old *WRKY75* mutant and overexpression lines at 4 days post-inoculation with *A. brassicicola* spores. Values are mean SE (n = 3 experiments), and asterisks indicate significant differences as compared to controls based on one way ANOVA (**P<0.01).

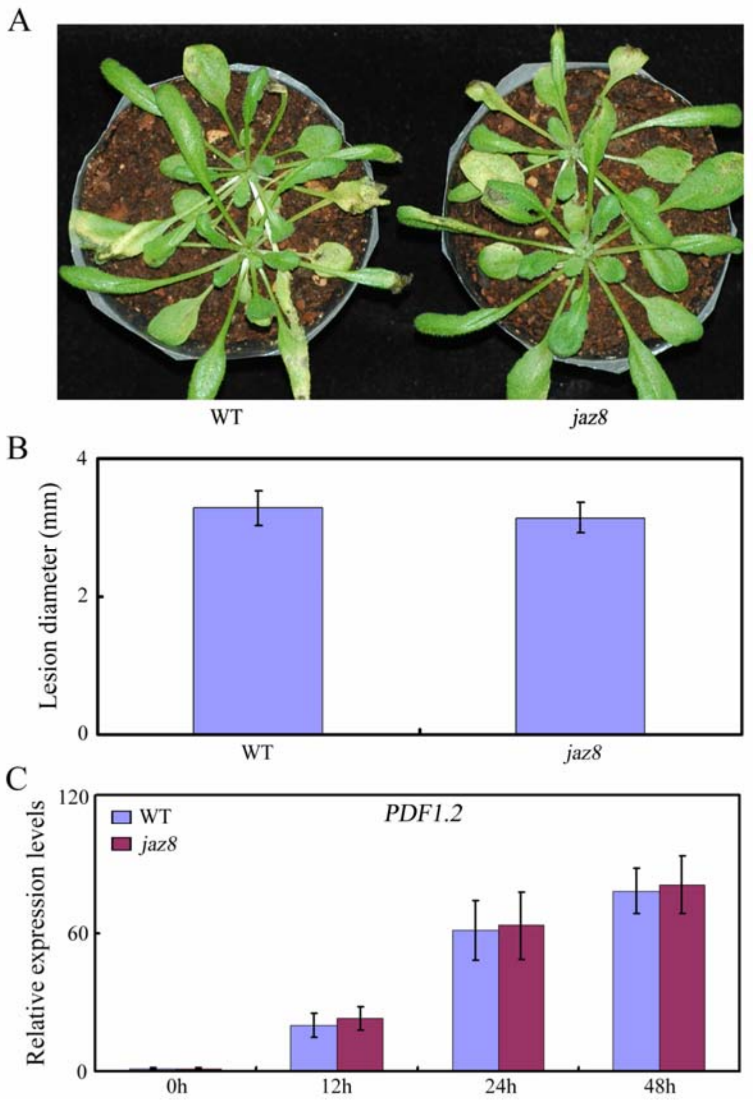

Figure S3. Phenotypic characterization of the *jaz8* mutant plants upon *B.cinerea* infection.

A. Disease symptom development. Leaves of the indicated genotypes were inoculated by spraying with a spore suspension of *B.cinerea*. Plants were maintained at high humidity and disease symptoms were photographed at 5 dpi.

B. The lesion sizes on detached rosette leaves from 5-week-old plants at 3 days post-inoculation with *B. cinerea* spores. Values are mean SE (n = 3 experiments).

C. Expression of *PDF1.2* in WT and *jaz8* after inoculation with *B.cinerea* for 0, 12, 24, and 48 h, respectively. *ACTIN2* and *UBQ5* were used as internal controls. Values are mean SE (n = 3 experiments).

Table S1: A checklist outlining the RNA to qRT-PCR quality/methodology.

Item to check	Details
Definition of experimental and control groups	Provided in the methods section.
Number within each group	At least twenty indivigual seedlings at indicated time upon Botrytis inoculation
Assay carried out by core lab or investigator's lab?	Investigator's lab
Description	Provided in the methods section
Volume/mass of sample processed	The plants was frozen immediately with liquid nitrogen, ground by using homogenizer. A 200 mg of the powder was used for RNA extraction.
Microdissection or macrodissection	
Processing procedure	
If frozen - how and how quickly?	
If fixed - with what, how quickly?	
Sample storage conditions and duration (especially for FFPE samples)	
Procedure and/or instrumentation	According to RNeasy Plus Mini Kit (Qiagen, Cat# 74134) manual. No modification and additional reagents were used.
Name of kit and details of any modifications	
Source of additional reagents used	
Details of DNase or RNAse treatment	Remove DNA contamination by using the gDNA eliminator spin column included in RNeasy Plus Mini Kit (Qiagen
Contamination assessment (DNA or RNA)	No amplification was detected in no RT samples.
Nucleic acid quantification	Spectrophotometer
Instrument and method	A 1:50 dilution was measured with the Eppendorf BioPhotometer D30 (Eppendorf)
Purity (A260/A280)	The A260/280 ratio is generally 1.8~2.0.
Yield	About 0.2~0.5 µg per 1 mg leaves
RNA integrity method/instrument	RNA integrity (RIN/RQI or Cq of 3' and 5' transcripts) was not confirmed. However, the RNA integrity was confirmed by 1.2% agarose gel electrophoresis.
RIN/RQI or Cq of 3' and 5' transcripts	
Electrophoresis traces	
Inhibition testing (Cq dilutions, spike or other)	No inhibition was confirmed by inhibition testing with Cq dilutions
Complete reaction conditions	According to PrimeScript II 1st strand cDNA Synthesis Kit (Takara Biotech, Kusatsu, Shiga, Japan) manual
Amount of RNA and reaction volume	20 µl reaction volume containing 1 µg of total RNA
Priming oligonucleotide (if using GSP) and concentration	2.5 µM of oligo dT primer included in PrimeScript II 1st strand cDNA Synthesis Kit (Takara Biotech)
Reverse transcriptase and concentration	10 U/µl of PrimeScript II RTase included in PrimeScript II 1st strand cDNA Synthesis Kit (Takara Biotech)
Temperature and time	42°C for 1 hour
Manufacturer of reagents and catalogue numbers	PrimeScript II 1st strand cDNA Synthesis Kit (Takara Biotech, Cat# 6210A)
Cqs with and without RT	The amplicon of *Actin2* and *UBQ5* were used for the evaluation of the cDNA quality and in this case the Cqs with RT are about 20-24, without RT no amplification was detected.
Storage conditions of cDNA	Stored at -20°C
If multiplex, efficiency and LOD of each assay.	Multiplex qPCR was not performed.
Sequence accession number	Shown in methods section
Location of amplicon	Exons
Amplicon length	among 120-200bp
In silico specificity screen (BLAST, etc)	BLAST, no pseudogenes or homologs were found.
Pseudogenes, retropseudogenes or other homologs?	
Sequence alignment	
Secondary structure analysis of amplicon	Not analyzed
Location of each primer by exon or intron (if applicable)	Exons
What splice variants are targeted?	No variant
Primer sequences	Shown in Additional table 2
RTPrimerDB Identification Number	None
Probe sequences	None
Location and identity of any modifications	None
Manufacturer of oligonucleotides	BGI TECH SOLUTIONS BEIJING LIUHE CO. LIMITED
Purification method	Salt-free
Complete reaction conditions	Provided in the methods section
Reaction volume and amount of cDNA/DNA	20 µl reaction volume containing 1 µl of cDNA reaction mixture
Primer, (probe), Mg++ and dNTP concentrations	200 nM of each forward and reverse primer
Polymerase identity and concentration	Included in the Power SYBR Green PCR Master Mix
Buffer/kit identity and manufacturer	TB Green® Premix Ex Taq™(Takara, Cat# RR820A)
Exact chemical constitution of the buffer	Included in the Power SYBR Green PCR Master Mix
Additives (SYBR Green I, DMSO, etc.)	SYBR Green I, included in the Power SYBR Green PCR Master Mix
Manufacturer of plates/tubes and catalog number	LightCycler 480 Multiwell Plate 96 (Cat# 4729692001)
Complete thermocycling parameters	95°C for 10 min, 40 cycles of 95°C for 15 sec and 60°C for 1 min, followed by dissociation step [95°C for 15 sec, 60°C for 30 sec and 95°C for 15 sec]
Reaction setup (manual/robotic)	Manual
Manufacturer of qPCR instrument	The LightCycler 480 II from Roche
Specificity (gel, sequence, melt, or digest)	The products were confirmed by size estimations on a 2% agarose gel and by analyzing their melting curves in the dissociation step.
For SYBR Green I, Cq of the NTC	No or negligible Cqs
Evidence of optimisation (from gradients)	The standard carves with slopes between -3.4 to -3.9 and y-intercepts between 38 to 40, when the log10 of the template concentration is plotted on *x*-axis and Cq is plotted on *y*-axis.
Standard curves with slope and y-intercept	
PCR efficiency calculated from slope	0.79 - 0.97
Confidence interval for PCR efficiency or standard error	Not analyzed
r2 of standard curve	>0.99
Linear dynamic range	Accurate quantification was possible over 6 orders of magnitude of concentration.
Cq variation at lower limit	Cq of 35
Confidence intervals throughout range	Not analyzed
Evidence for limit of detection	Cq values obtained after cycle 40 were considered out of accurate quantifiable range.
If multiplex, efficiency and LOD of each assay.	Multiplex qPCR was not performed.
qPCR analysis program (source, version)	Applied Biosystems, SDS v1.4
Cq method determination	Setting the threshold to 0.2
Outlier identification and disposition	No data have been excluded from the calculations.
Results of NTCs	No or negligible Cqs
Justification of number and choice of reference genes	The *Actin2* and *UBQ5* were used as references.
Description of normalisation method	
Number and concordance of biological replicates	3
Number and stage (RT or qPCR) of technical replicates	To check reprducibility, we performed qPCR of both *Actin2* and *UBQ5* with 4 or 5 technical replicates in the cDNA samples used in this study. The results showed that the difference in Cqs was confirmed to be between 0.3-0.7 (0.5-1.1%CV) among the qPCR replicates. Moreover, the expression levels of the genes analyzed were confirmed to be similar between 3 biological replicates.
Repeatability (intra-assay variation)	
Reproducibility (inter-assay variation, %CV)	
Power analysis	We did not perform statistical analysis. However, the expression levels of the genes analyzed were confirmed to be similar between 3 biological replicates.
Statistical methods for result significance	
Software (source, version)	
Cq or raw data submission using RDML	None

Table S2. Primers used in this study.

Use	Primers (5'->3')	
BiFC Assay	WRKY75-YC-1:	TATCTAGAATGGAGGGATATGATAATGGG
	WRKY75-YC-2:	TAGGATCCGAAAGAAGAGTAGATTTGCATTTGA
	JAZ4-YN-1:	CAACATTTAAATATGGAGAGAGATTTTCTCGG
	JAZ4-YN-2:	AAATCTAGAGTGCAGATGATGAGCTGG
	JAZ8-YN-1:	AAAATTTAAATATGAAGCTACAGCAAAATTGTG
	JAZ8-YN-2:	AAATCTAGATCGTCGTGAATGGTACGGTGAAG
qRT-PCR analysis of gene expression	WRKY75-q1:	ATATGGCCAAAAGGCCGTCA
	WRKY75-q2:	TGCTCGAAGTTTTCGGTGGA
	ORA59-q1:	AAAAGAAGAAGGAAAAGAAGCCAC
	ORA59-q2:	GTGTCGAATGTCCCAAGCCA
	PDF1.2-q1:	TCACCCTTATCTTCGCTGCTCT
	PDF1.2-q2:	ATGATCCATGTTTGGCTCCTTC
	JAZ8-q1:	CTCAAACGGGTCGGATCCTC
	JAZ8-q1:	CGTCGTGAATGGTACGGTGA
	ACTIN2-q1:	TGTGCCAATCTACGAGGGTTT
	ACTIN2-q2:	TTTCCCGCTCTGCTGTTGT
	UBQ5-q1:	GTTAAGCTCGCTGTTCTTCAGT
	UBQ5-q2:	TCAAGCTTCAACTCCTTCTTTC
qRT-PCR amplification of *ORA59* promoter	ORA59-W-box123-1:	CCATGTCACATGTGTATCAGA
	ORA59-W-box123-2:	TAATAGAATAACACACTGAAGATGGT
	ORA59-W-box4-1:	GGTTAGGCTAGGGACGATAG
	ORA59-W-box4-2:	GTACTCTCCATGCATTATCACTTAT
	ORA59-W-box567-1:	GAAATCTCAGCCAATTCAACT
	ORA59-W-box567-2:	CCTAGAGCAATCACAAACTAATG
	ORA59-W-box8-1:	ACTTGAATGTGAAGACGTTGA
	ORA59-W-box8-2:	GATGTTACCATCACAACAGTTG
	ORA59-W-box9-1:	TATAATATAATGGAATCATTCAGTCG
	ORA59-W-box9-2:	GTTGTTAAACGTACCACTTACCA

Table S3. *WRKY* genes screened in this study.

3	At2g03340	Salk_107019
4	At1G13960	Salk_073118
6	At1g62300	Salk_012997
9	At1g68150	Salk_067122
10	At1g55600	Salk_050364
11	At4g31550	Salk_141511
16	At5g45050	Salk_001360
17	At2g24570	Salk_094997
18	At4g31800	Salk_093916
19	At4g12020	Salk_052230
20	At4g26640	Salk_055904
22	At4g01250	Salk_094892
24	At5g41570	Salk_008183
25	At2g30250	Salk_136966
26	At5g07100	Salk_003386
32	At4g30930	Salk_091352
33	At2g38470	Salk_006603
34	At4g26440	Salk_133019
38	At5g22570	CS858252
39	At3G04670	SALK_073483C
40	At1g80840	ET5883
41	At4g11070	Salk_068648
45	At3G01970	GK-684G12-023118
46	At2g46400	Salk_134310
47	At4g01720	Salk_052594
48	At5g49520	Salk_066438
49	At5g43290	Salk_091556
51	At5g64810	Salk_022198
52	At5g45270	Salk_099734
53	At4g23810	Salk_034157
54	At2g40740	Salk_046765
55	At2g40750	Salk_111964
56	At1g64000	Salk_130727
58	At3g01080	Salk_036220
59	At2g21900	Salk_039436
60	At2g25000	Salk_120706
63	At1g66600	Salk_007496
64	At5g13080	Salk_101367
66	At5g15130	Salk_055293
68	At3g62340	Salk_000087
70	At3g56400	Salk_025198
WRKY28 RNAi	At4G18170	

WRKY45 RNAi	At3G01970	
WRKY71 RNAi	At1G29860	
WRKY75 RNAi	At5G13080	
